# Impact of Social Media Usage on Users’ COVID-19 Protective Behavior: Survey Study in Indonesia

**DOI:** 10.2196/46661

**Published:** 2023-04-13

**Authors:** Putu Wuri Handayani, Guilherme Augusto Zagatti, Hajer Kefi, Stéphane Bressan

**Affiliations:** 1 Information Systems Undergraduate Study Program University of Indonesia Depok Indonesia; 2 Institute of Data Science, National University of Singapore Singapore Singapore; 3 Digital Data Design, Paris School of Business France France; 4 School of Computing, National University of Singapore Singapore Singapore

**Keywords:** COVID-19, pandemic, infectious diseases, social media, trust, behavior, Indonesia

## Abstract

**Background:**

Social media have become the source of choice for many users to search for health information on COVID-19 despite possible detrimental consequences. Several studies have analyzed the association between health information–searching behavior and mental health. Some of these studies examined users’ intentions in searching health information on social media and the impact of social media use on mental health in Indonesia.

**Objective:**

This study investigates both active and passive participation in social media, shedding light on cofounding effects from these different forms of engagement. In addition, this study analyses the role of trust in social media platforms and its effect on public health outcomes. Thus, the purpose of this study is to analyze the impact of social media usage on COVID-19 protective behavior in Indonesia. The most commonly used social media platforms are Instagram, Facebook, YouTube, TikTok, and Twitter.

**Methods:**

We used primary data from an online survey. We processed 414 answers to a structured questionnaire to evaluate the relationship between these users’ active and passive participation in social media, trust in social media, anxiety, self-efficacy, and protective behavior to COVID-19. We modeled the data using partial least square structural equation modeling.

**Results:**

This study reveals that social media trust is a crucial antecedent, where trust in social media is positively associated with active contribution and passive consumption of COVID-19 content in social media, users’ anxiety, self-efficacy, and protective behavior. This study found that active contribution of content related to COVID-19 on social media is positively correlated with anxiety, while passive participation increases self-efficacy and, in turn, protective behavior. This study also found that active participation is associated with negative health outcomes, while passive participation has the opposite effects. The results of this study can potentially be used for other infectious diseases, for example, dengue fever and diseases that can be transmitted through the air and have handling protocols similar to that of COVID-19.

**Conclusions:**

Public health campaigns can use social media for health promotion. Public health campaigns should post positive messages and distil the received information parsimoniously to avoid unnecessary and possibly counterproductive increased anxiety of the users.

## Introduction

Since their inception a decade or so ago, social media platforms have steadily increased their prevalence as substitutes for face-to-face interactions and other existing forms of communication. From the start of the pandemic social media have become the main virtual agora where news and opinions about COVID-19 have been posted, shared, and commented on by public and private organizations and by individuals [1]. Digital social media seem to confirm only further that “the medium is the message” [2]. The effect of the printing press on the Enlightenment [2] or of social media on surveillance capitalism [3] demonstrates the power of the medium in shaping society. Research is challenged to evaluate “the change of scale or pace or pattern [intro-duced] into human affairs” [4] and, ultimately, the effects that digital social media exert on society, in particular, in a situation such as that of the COVID-19 pandemic.

This study presents an analysis of the effects of social media usage on adaptive responses to epidemics. In times of uncertainty, users contribute or consume social media content, which could act as a catalyst for anxiety, in turn triggering protective action [5]. Alternatively, users might engage with social media to reduce anxiety levels as they can relate with others and find useful information to protect themselves. Social media could also affect the belief in the best course of action or self-efficacy. Moreover, trust in social media platforms could modulate the belief in the truthfulness of their contents affecting all the aforesaid mechanisms. Our research focuses on measuring the relationship between the consumption and production of social media content, trust in social media, anxiety, self-efficacy, and adaptive behavior in Indonesia.

The COVID-19 situation as a pandemic was first described on March 11, 2020, and in early 2020, the first 2 cases of COVID-19 in Indonesia had been confirmed [6]; thus, restrictions on gathering and movement were implemented by the Indonesian government. Of its 272 million inhabitants, around 56% live on the island of Java [7]. The first case of COVID-19 in Indonesia was reported on March 2, 2020. As of July 2022, Indonesia recorded close to 160 million COVID-19 deaths, placing it in the top 10 countries with the most casualties [8].

From the second half of 2021 to the closure of our study, Indonesia ranked worldwide in the top quantile of the Stringency Index, a composite of containment, closure, and health system policy indicators comparable across countries [9]. By the end of our study, the Indonesian government still imposed restrictions on the population using the Community Activity Restrictions Enforcement policy [8]. The country counted with recommended closure of schools, workplaces, and public transport; recommended cancelation of events; and recommended restriction of internal movement. There were coordinated public health campaigns, but contact tracing was limited. International borders were closed, and vaccination was made compulsory.

Kemp [10] reported that social media penetration in Indonesia increased from 59% of the population in January 2020 to 68.9% at the beginning of 2022, with average daily usage close to 4 hours. In 2021, YouTube (Google LLC) and Instagram (Meta Platforms, Inc.) reached approximately 94% and 87% of internet users, respectively, and college students were the most active on social media [10]. The Indonesian government used multiple social media channels, including Facebook (Meta Platforms, Inc.), Instagram, Twitter (Twitter, Inc.), YouTube, and TikTok (ByteDance), to conduct its COVID-19 health campaigns.

Previous research on health information in social media is limited to the analysis of secondary data, especially social media content with a specific theme for certain disease/health information, and did not focus on COVID-19–related information. Tsao et al [1] reviewed COVID-19 and social media and found that most research focuses on surveying public attitudes toward COVID-19. Huesch et al [11] analyzed maternity care campaigns from Twitter, Facebook, and Google data. Ahmed and Rasul [12] described that increased social media usage is associated with believing and sharing COVID-19 misinformation. Chen et al [13], Lwin et al [14], and Buchanan et al [15] analyzed the Twitter data set regarding COVID-19–related information.

Moreover, Wang et al [16] found that pregnant women use social media to search for COVID-19 information and social media use was directly associated with depression. Several studies have examined users’ intention to seek health information on social media [17] and the impact of social media use on mental health in Indonesia [18,19]. Sujarwoto et al [18] showed that social media harm adult mental health. Maurizka et al [19] also found that social media content influences depressive symptoms among the younger generation in Indonesia. Sujarwoto et al [18] reported that university students experienced mild depression when they frequently used social media during the COVID-19 pandemic in Indonesia. Future studies should examine the benefits of using social media to facilitate knowledge sharing for other health stakeholders, such as social media users [20]. To address these research gaps, this study analyzes the impact of social usage on COVID-19–protective behavior in Indonesia. This study also proposes a conceptual model that describes the impact of social media usage on COVID-19 protective behavior in Indonesia.

The ability of social media to influence human behavior has promoted them to the role of media of choice for modern marketing [3], political [21], and public health campaigns [22,23]. It is already the case in many other countries that public health officers use both traditional and social media to run public health campaigns. Social media not only serve as media to disseminate messages quickly and effectively to the public but also reach journalists and specialists and shape the public discourse about health. Huesch et al [11] ran a pilot public health campaign on 3 social media platforms and reported that they could be cost-effective in reaching a targeted audience. The authors remarked that the effectiveness of the campaigns might depend on the availability of resources and the experience of health officers.

Negative emotions, such as worry and fear, seem to be powerful nudges [24] leveraged in many public health campaigns [25-28]. By describing the terrible predicaments that an individual could face, health campaigns trigger individuals to take actions [26,27]. The communication media amplify emotions and nudges by increasing exposition to the message and its salience. The images and videos broadcast by television channels and social media are literally and metaphorically graphic depictions of the pathology and other consequences of the disease. Worry and fear were commonly expressed on social media during the pandemic. Not only morbidity risks trended in social media, but also socioeconomic concerns related to the policies implemented to combat the virus [1].

Fear can be particularly effective when paired with high levels of self-efficacy, that is, the belief in one’s ability to execute certain behaviors to achieve the desired goal. Fear-inducing messages that carry high efficacy, for example, “COVID-19 is highly contagious, but can be avoided by wearing a face mask,” tend to promote protective behavior compared with those with low efficacy, which tend to be rejected by the receiver [25]. In the context of the Zika epidemics in the United States in 2016, the volume of social media messages circulated in the community was positively associated with increased risk perceptions captured from a longitudinal survey representative of the American population [28]. However, traditional media such as print and TV were more strongly correlated with higher levels of protective behavior, suggesting some complementarity between both media types.

Studies using convenient samples have also found that fear and self-efficacy mediate the path from media usage to protective behavior. Zhang et al [27] investigated the H1N1 epidemics in the United States in 2009, while Mahmood et al [29] and Liu [30] looked at the COVID-19 epidemic in 2020 in Pakistan and China, respectively. These 3 studies have a similar design. They used an online survey, recruited between 300 and 500 participants, and employed structural equation modeling to analyze their data. However, each study refers to self-efficacy differently; for example, Zhang et al [27] used self-knowledge, Mahmood et al [29] used self-efficacy, and Liu [30] used self-responsibility. In addition, Liu [30] surveyed media usage generally, whereas the remaining 2 papers focused exclusively on social media.

In contrast to the previous 3 studies, Yoo et al [31] split social media usage into passive and active participation to identify how social media usage affects protective behavior through 2 distinct paths. On the one hand, they found that passive participation in social media is associated with an increase in perceived threat and has a direct positive effect on protective behavior. However, the effect on self-efficacy was not statistically significant. On the other hand, active participation is positively related to self-efficacy and has a direct negative effect on protective behavior, but there is no effect on perceived threat.

Given the findings observed in the aforementioned studies, we would like to confirm whether a similar hypothesis also holds in our empirical setting, namely:

- H1: Active contribution of COVID-19 content in social media will be positively associated with anxiety (H1a) and self-efficacy (H1b) to COVID-19.
- H2: Active contribution of COVID-19 content in social media will be positively associated with protective behavior against COVID-19.
- H3: Passive consumption of COVID-19 social media content will be positively associated with anxiety (H3a) and self-efficacy (H3b) to COVID-19.
- H4: Passive consumption of COVID-19 social media content will be positively associated with protective behavior against COVID-19.
- H5: Anxiety (H5a) and self-efficacy (H5b) to COVID-19 will be positively associated with protective behavior against COVID-19.

Gibson and Trnka [32] described trust as receiving or giving support to their peers on social media; in their study, youth held the highest level of trust toward health-related information on social media [33]. Huh et al [34] showed that, for example, the higher the level of trust in online drug-related information, the more likely one would engage in the protective behaviors such as seeking more health-related information. Not only the youth, but also the government used social media to communicate with the public during COVID-19 [35]. The government’s communication alongside its response to COVID-19 enhanced the public’s trust.

Therefore, trust plays a crucial role in social media; thus, this variable must be studied by academics [36,37]. Based on Kožuh and Čakš [38], social media is one of the credible sources of information in a health crisis. In addition, according to Jin et al [39], user’s willingness to share and adopt health knowledge is dependent on their trust in social media. The more users engage with social media news, the more they trust the actual situation around COVID-19 [38], thereby potentially increasing their self-efficacy and protective behavior. Moreover, trust in social media could influence users’ response to comply with COVID-19 protocol guidance [40].

Compared with traditional media, social media allow the fast spread of information and the publication of less trustworthy materials in which both the veracity of the message and the intention of the publisher are challenging to verify. Social media platforms are also responsible for mediating the content delivered to their users using automated mechanisms not open to scrutiny. Increased social media activity can contribute to the fast spread of false information [41,42] that can decrease social morals and increase levels of anxiety [43]. Overall, these affordances could affect trust in social media, which, in turn, could affect all the variables under investigation in this study.

We thus consider the following hypothesis:

- H6: Trust on social media is positively associated with the active contribution of COVID-19 content in social media (H6a), and the passive consumption of COVID-19 social media content (H6b).
- H7: Trust on social media is positively associated with anxiety (H7a) and self-efficacy (H7b) to COVID-19.
- H8: Trust on social media positively affects protective behavior against COVID-19.

**Figure 1:**
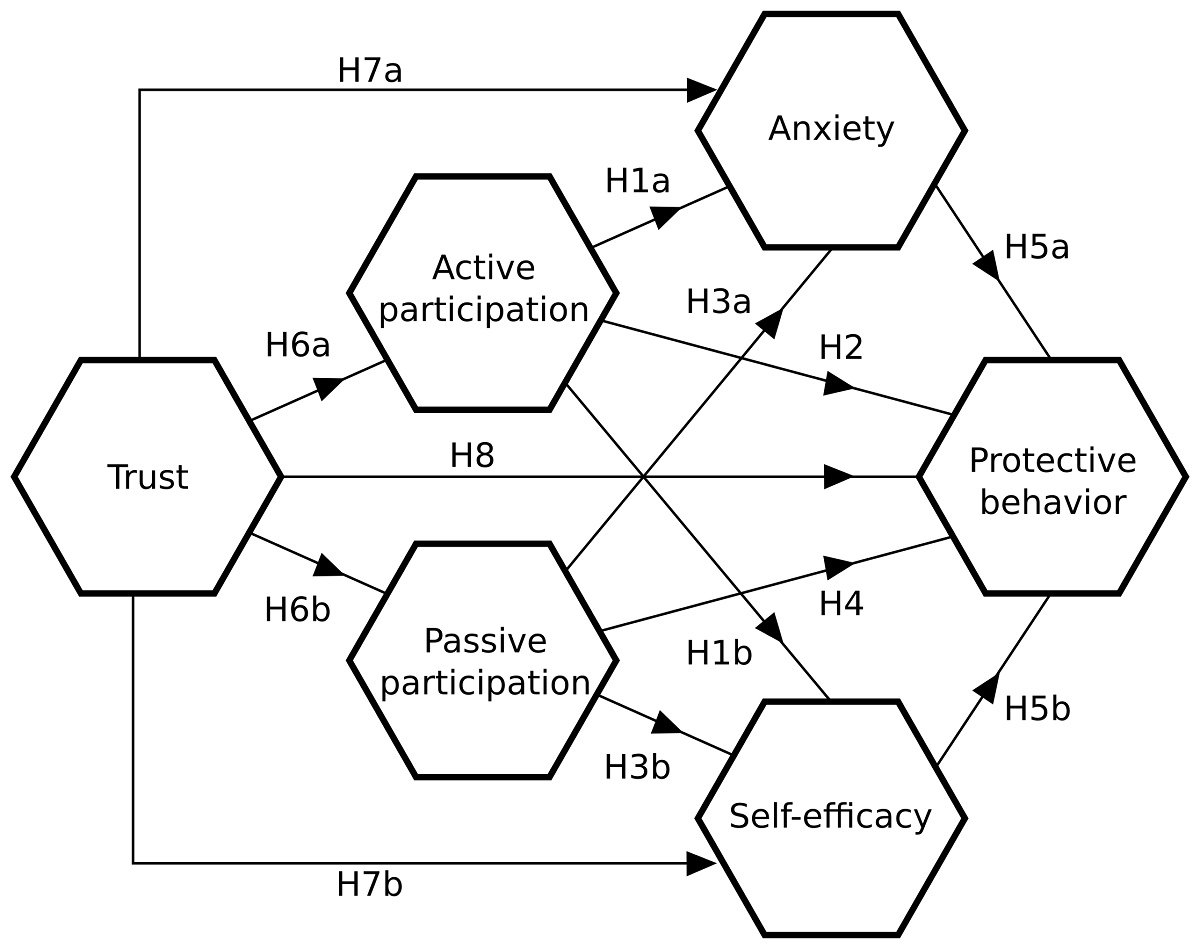
Hypothesized relationship between constructs.

We can express these relationships as a direct acyclic graph, depicted in Figure 1.

## Methods

### Research Model

We propose and define 6 constructs in our proposed model: active participation, passive participation, anxiety, self-efficacy, protective behavior, and trust.

First and second, we propose “active participation” and “passive participation” to qualify the engagement of social media users, respectively. The former refers to the publication of COVID-19 content on social media, whereas the latter to its reception and consumption. These constructs follow the discussion in Chen et al [13] and Yoo et al [31]. Users are likely to blend different forms of engagement, from more active ones such as forwarding, replying to, and creating posts to more passive ones such as reading or “liking” a post.

Third, we consider “anxiety,” defined as the feeling of tension, worried thoughts, and physical changes aroused when reading COVID-19 materials on social media.

Fourth, we define self-efficacy as an individual’s belief in his/her ability to protect herself/himself against COVID-19 [25].

Fifth, modifying Yoo et al [31], protective behavior refers to the adoption of behaviors deemed beneficial to COVID-19 prevention.

Finally, we define trust as a firm belief in the integrity of social media platforms and their content.

The 6 constructs were measured in terms of reflective indicators as captured in responses to questions and are listed in Multimedia Appendix 1. We defined the measurement items according to Chen et al [13], Chan et al [28], Mahmood et al [29], Yoo et al [31], Plohl and Musil [40], Liu and Liu [44], Padidar et al [45], and World Health Organization [46].

All responses were collected on a Likert scale. Responses to questions meant to quantify active participation and passive participation used a Likert scale with the following subjective frequency levels: 5=very frequently, 4=frequently, 3=sometimes, 2=rarely, and 1=never. Questions quantifying the 4 other constructs used Likert scales with the following agreement levels: 5=strongly agree, 4=agree, 3=neutral, 2=disagree, and 1=strongly disagree.

We then used structural equation modeling to test the hypotheses [47,48]. Structural equation modeling is a technique that simultaneously models the relationships between composite items and their constructs and the relationships between constructs. All hypothesis relationships are modeled as linear regressions. Structural equation techniques differ according to the estimation procedure [49]. This study used partial least square path modeling to fit the model to the data, which uses a variance-based optimization criterion with limited information [47]. In essence, it consists of iteratively fitting linear regressions until the coefficients of each of the regressions converge between iterations.

### Research Procedure

We used an online survey approach driven by the COVID-19 pandemic, as this is expected to reach wider respondents. The questionnaire was prepared in Indonesian language with the expectation that the respondents will easily understand the questions. Prior to the survey distribution, we carried out a pilot study involving 30 respondents who have actively used social media. The Cronbach α value for each variable was above .7. Moreover, we conducted an online survey from February 28, 2022, to March 28 2022. The respondents were all Indonesian residents. The survey instrument was prepared in Indonesian and contained 50 questions organized into 4 sections corresponding to demographics, frequency of social media participation, perceptions of COVID-19 social media content, and additional details on social media behavior. Links to the survey were distributed through popular social media platforms in Indonesia, such as Instagram, Facebook, Twitter, WhatsApp, Telegram, and Line (NHN Japan [now Line Corporation]). Respondents were encouraged to share the links to the survey with their social networks; thus, we used snowball sampling to make it easier to find trusted respondents from the network of friends that each respondent has. The age of users ranged from 18 to 34 years, and comprised approximately 65% (~110 million) of the total active users of social media [10]. In total, we obtained 416 complete responses, 2 of which were discarded as they had been submitted by respondents not residing in Indonesia in the period of interest.

### Ethical Considerations

This study has received approval from the Faculty of Computer Science, University of Indonesia and all respondents have agreed to participate in this study. No ethics board review was sought as all respondent data are anonymous and the data can only be used for the purposes of this research.

## Results

### Respondent Demographics

The survey responses were evenly distributed across gender. There was a 2-peaked income distribution with mass at both extremes (<IDR 3 million and >IDR 7 million; IDR 1=US $0.000066); 7 out of 10 respondents resided in Jakarta (the capital city of Indonesia). More than one-half of the respondents were under the age of 30 and undergraduate students. According to Kemp [10], 93.8% of Indonesians view/post content on YouTube, 86.6% use Instagram, and 85.5% post on Facebook. While most respondents used Instagram and YouTube, Facebook, TikTok, and Twitter were much less popular; 68.4% (283/414) of respondents followed accounts from news organizations, and only a minority followed accounts from government or private organizations. A vast majority sought influencers’ content and health information. Table 1 presents a summary of the respondents’ demographics.

**Table 1:** Respondents demographics (N=414).

Demographics				Values, n (%)
**Respondent characteristics**				
	**Gender**			
		Male	204 (49.3)	
		Female	210 (50.7)	
	**Income (in millions IDR^a^)**			
		<3	161 (38.9)	
		3-5	51 (12.3)	
		5-7	48 (11.6)	
		>7	154 (37.2)	
	**Region**			
		Jakarta	279 (67.4)	
		Java (excluding Jakarta)	64 (15.5)	
		Sumatera	20 (4.8)	
		Kalimantan	19 (4.6)	
		Others	32 (7.7)	
	**Age**			
		<17	4 (1.0)	
		17-27	218 (52.7)	
		28-38	115 (27.8)	
		39-49	60 (14.5)	
		>50	17 (4.1)	
**Social media consumption (multiple choices allowed)**				
	**Platforms**			
		Instagram	347 (83.8)	
		Facebook	146 (35.3)	
		YouTube	302 (72.9)	
		TikTok	112 (27.1)	
		Twitter	166 (40.1)	
	**Accounts followed**			
		Government	137 (33.1)	
		News organizations	283 (68.4)	
		Private organizations	115 (27.8)	
	**Content consumed**			
		Influencers	279 (67.4)	
		Health statistics	162 (39. 1)	
		Users’ comments	212 (51.2)	
		Health information	287 (69.3)	

^a^IDR 1=US $0.000066.

### Structural Equation Modeling

The number of items k used for each construct and their internal consistency as measured by Dijkstra and Henseler ρA are displayed in Table 2. All constructs are internally consistent, attaining ρA well above the recommended value of 0.7 [47]. Further, all pairs of constructs displayed a heterotrait-monotrait ratio below the recommended amount of 0.85 as shown in Table 3. The heterotrait-monotrait ratio is a measure of discriminant validity to check whether constructs are not equivalent. It is constructed as the ratio between the mean correlation of constructs across indicators and the mean correlation of constructs within indicators [47].

Hypotheses H1-H8 are represented in terms of the direct acyclic graph (Figure 1). Each node represents a dependent variable in a linear regression in which the sources of its incoming edges are the independent variables. To estimate the model, we standardized each variable such that its mean is equal to 0 and the SD is equal to 1. The estimated coefficients are displayed in Table 3. The table also displays SEs that are computed via bootstrapping with 5000 repetitions; the significance level presented is based on the assumption that the *t*-statistics coefficient divided by SE approximates a standard normal distribution.

**Table 2:** Hypothesis testing results^a^.

Results	Reliability				Regressions^b^				
	k	ρA	AVE^c^	Active participation		Passive participation	Anxiety	Self-efficacy	Protective behavior
Active participation	4	0.917	0.770	N/A^d^		N/A	0.245^e^; 0.058; *P*<.001; H1a ✓	0.034; 0.045; *P*=.45; H1b	–0.210^e^; 0.062; *P*<.001; H2
Passive participation	3	0.767	0.677	N/A		N/A	–0.023; 0.053; *P*=.67; H3a	0.270^e^; 0.051; *P*<.001; H3b ✓	0.085; 0.060; *P*=.15; H4
Anxiety	7	0.937	0.689	N/A		N/A	N/A	N/A	–0.064; 0.044; *P*=.15; H5a
Self-efficacy	4	0.879	0.721	N/A		N/A	N/A	N/A	0.207^e^; 0.053; *P*<.001; H5b ✓
Trust	4	0.905	0.781	.272^e^; 0.048; *P*<.001; H6a ✓		.413^e^; 0.039; *P*<.001; H6b ✓	0.123^f^; 0.054; *P*=.02; H7a ✓	0.359^e^; 0.053; *P*<.001; H7b ✓	0.246^e^; 0.053; *P*<.001; H8 ✓
Protective behavior	6	0.867	0.581	N/A		N/A	N/A	N/A	N/A
*R* ^2^				0.074		0.170	0.085	0.296	0.170
Akaike information criterion				–28.8		–74.4	–29.8	–138.6	–66.3
Total number of observations (N)				414		414	414	414	414

^a^The table reports reliability tests on the first 3 columns: k is the number of items in each construct; Dijkstra and Henseler ρA is a measure of internal consistency; AVE is a measure of convergence validity. The last 5 columns display the coefficients of the fitted linear regressions for the model displayed in Figure 1 with significance-level indicators based on the assumption that the *t*-statistics approximates a standard normal distribution.

^b^After each regression coefficient, the bootstrapped SEs are reported, followed by the P values and the corresponding hypothesis. The hypotheses that are validated by our data are marked with ✓.

^c^AVE: average variance extracted.

^d^N/A: not applicable.

^e^*P*<.001.

^f^*P*<.05.

**Table 3:** Result of heterotrait-monotrait ratio.

Measure	Active participation	Passive participation	Anxiety	Self-efficacy	Protective behavior
Passive participation	0.49	N/A^a^	N/A	N/A	N/A
Anxiety	0.28	0.15	N/A	N/A	N/A
Self-efficacy	0.27	0.52	0.27	N/A	N/A
Protective behavior	0.12	0.23	0.09	0.34	N/A
Trust	0.30	0.49	0.20	0.54	0.35

^a^N/A: not applicable.

The average variance extracted (AVE) for all fitted constructs is above the recommended level of 0.5 [47]. The AVE can be interpreted as the average loading on each construct. Lower AVEs suggest that some constructs might have weak item loading. Table 3 shows that “protective behavior” has a loading of 0.581, which might indicate a lower agreement between its composite items. However, due to the disparate nature of protective measures ranging from frequent handwashing to vaccination, it is reasonable to expect a lower AVE.

Concerning the fitted coefficients, we observed an agreement with hypothesis H1a, which posits a positive relationship between passive social media participation and anxiety. By contrast, hypotheses H1b (*P*=.45) and H2 (*P*=.001) do not agree with the data as there is no statistically significant relationship between social media contribution and self-efficacy and there is a negative relationship with protective behavior. Next, hypothesis H3b is satisfied, whereas H3a (*P*=.67) and H4 (*P*=.15) are not. There is a positive association of passive social media participation with self-efficacy, but no significant association with either anxiety or protective behavior. We also observed an agreement with hypothesis H5b, which posits an association between self-efficacy and protective behavior. Taken together, the previous 2 results suggest that self-efficacy works as a mediator between social media consumption and protective behavior. By contrast, we observed no statistically significant relationship between anxiety and protective behavior invalidating hypothesis H5a (*P*=.15).

Moreover, we wanted to uncover the roles that trust in social media could play on the other variables. Indeed, we found that trust is positively and significantly associated with all of our constructs in agreement with hypotheses H6a (*P*<.001), H6b (*P*<.001), H7a (*P*=.02), H7b (*P*<.001), and H8 (*P*<.001). However, there are notable differences. In particular, the coefficients of “trust” on “passive participation” and “self-efficacy” are close to 0.4 of an SD, which indicates that higher degrees of trust have a strong association with the use of social media as a medium of information acquisition. The coefficients of “trust” on “active participation” and “protective behavior” are smaller compared with the previous 2. By contrast, trust is only weakly associated with anxiety.

As we observed a lack of relationship between the constructs “active participation” and “self-efficacy,” we fitted an alternative model in which the causality between those variables is reversed, and there is a direct path between “passive participation” and “active participation.” In this alternative model, we found a statistically significant coefficient between “passive participation” and “active participation” equal to 0.354 (*P*<.001). The relationship between “active participation” and “self-efficacy” remained insignificant (*P*=.47) and there were no changes to the fit of the other dependent constructs.

With 6 constructs under investigation, there are just above 3 million possible networks. It is thus computationally feasible to evaluate the fit of all possible models defined as a direct acyclic graph. As a robustness check, we investigated whether our proposed model could capture most of the variation in the data as compared with alternatives based on the Akaike information criterion (AIC). Although seldom used for exploratory analysis, structural equation modeling is suited for this sort of exercise [49]. Neither the AIC nor any other similar measure was used as a selection criterion, and this exercise was only meant to compare the performance of our model with all other alternatives to provide some perspective.

The AIC for our model is presented in Table 3. The main target of our research is explaining “protective behavior” with regard to social media usage. We find that our proposed model for “protective behavior” falls in the 95th percentile of the AIC distribution for all models for this variable, which obtains a minimum of –75.1. The AIC penalizes models with a larger number of variables. However, given that most of the covariates for “protective behavior” are significant as displayed in Table 3, we obtain a strong fit for this variable. Besides, the AIC for self-efficacy and protective behavior is in the 75th percentile. Conversely, the fit for active and social media consumption falls in the 25th percentile, which is expected because we did not focus on explaining these variables.

## Discussion

### Principal Findings

This study shows that passive and active participation in social media has diametrically opposite effects. On the one hand, the active contribution of COVID-19 content in social media is detrimental to public health outcomes because it is associated positively with anxiety, negatively with protective behavior, and does not affect self-efficacy. Our results align with Yoo et al [31], who also found a negative direct relationship between active participation and protective behavior in South Korea. Although Liu and Liu [44] modeled the reverse directionality between active participation and anxiety, they also found a positive relationship between anxiety and active participation in China. Anxious participants in Belgium used social media more often as a coping mechanism for COVID-19 [50], and Belgium adolescents used social media to deal with anxious feelings during the COVID-19 quarantine period [50]. O’Day and Heimberg [5] found that people with social anxiety search social support from social media due to the lack of in-person support. In addition, based on Tsao et al [1], positive social media content linked to positive behavioral shifts of social media users, such as stay home and social distancing content messages, in this study.

On the other hand, this study finds that passive participation in social media was positively associated with public health outcomes. We identified a strong positive relationship between passive participation and self-efficacy, but no statistically significant relationship between anxiety and protective behavior. Appropriate practices toward COVID-19 are influenced by user’s good COVID-19 knowledge [51]. Thus, users who remained up to date with COVID-19 information through social media and other online channels will protect themselves [45]. Liu and Liu [44] described social media as a vital information channel that might positively influence people’s preventive behaviors. According to Shiloh et al [52], providing coping information and increasing behavior efficacy beliefs can effectively mobilize the public’s adoption of protective behaviors during COVID-19. However, this study presented results different from Zhang et al [27] in China and Mahmood et al [29] in Pakistan, which found that passive participation in social media influences anxiety and protective behavior.

Finally, this study shows that social media trust is a crucial antecedent where trust in social media is positively associated with active contribution and passive consumption of COVID-19 content in social media, users’ anxiety, self-efficacy, and protective behavior. Plohl and Musil [40] described that users’ trust in the entire social network and a member of the network is critical to their decisions to share knowledge in social networks. Therefore, users will choose social media where they find a network of trusted friends.

When trusted friends post messages concerning the efficacy or impact of various COVID-19 vaccines, personally connected people consider those messages with care. According to our study, the more trust users have in COVID-19 content on social media, the more protective behaviors they deploy. Therefore, social media trust indeed influences users’ compliance with COVID-19 protocol guidance [40]. Trust in others or social media peers (ie, governments, citizens, and news organizations) has been identified as a significant predictor of the willingness to cooperate, and trust toward fellow citizens is associated with prosocial behavioral intentions [53].

### Implications

This study analyzes the effects of social media usage on user’s protective behavior against the COVID-19 epidemic in Indonesia, the fourth most populous country in the world. This study also investigated both active and passive participation in social media, shedding light on cofounding effects of these different forms of engagement. Moreover, this study analyzed the role of trust in social media platforms and its effect on public health outcomes. Thus, this study enriches the study of Wijayanti et al [17], Sujarwoto et al [18], and Maurizka et al [19]. This study also adds to the research study of Wang et al [16], which only focused on pregnant women searching for COVID-19 information. This study also considered the perspective of social media users, thus enriching the study of Huesch et al [11], Ahmed and Rasul [12], Chen et al [13], Lwin et al [14], and Buchanan et al [15], all of which only analyzed the secondary data from social media posts.

This study provides practical implications for public health campaigns and social media users. Public health campaigns should use social media to post health promotion content to make social media users more aware of protecting themselves from infectious diseases. Social media users should also be aware of their active participation on social media with the hope of releasing their anxieties. Passive participation on social media was positively associated with public health outcomes. Therefore, social media users should consume health information from credible sources such as news, governments, or peers’ social media accounts. Social media providers should also filter their content to increase their users’ trust. Social media providers, together with public health campaigns, should provide awareness and knowledge about how to filter credible messages to social media users. Currently, the number of COVID-19–positive cases is starting to decrease; accordingly, the results of this study can potentially be used for other infectious diseases that have handling characteristics like COVID-19, for example, for dengue fever and others that can be transmitted by air and have handling protocols similar to COVID-19.

### Limitations

The respondents in this study are mostly located in the greater Jakarta region and may not faithfully reflect the diversity of the population of Indonesia. Then, although there likely exist feedback loops between some investigated constructs, we refrain from modeling such loops because of limitations in structural equation modeling. For instance, higher levels of active participation may lead to higher levels of passive social media participation because those users that post content are likely to spend more time reading reactions to their material. The challenge with feedback loops is that they can be hard to identify statistically. Dijkstra and Henseler [54] proposed the consistent partial least square estimator, which uses 2-stage least squares to identify endogenous effects. Identifying feedback loops using this methodology requires additional assumptions on the coefficients of the structural model, which we leave for future work.

### Conclusions

Opposite effects for active and passive participation in social media are found in this study. When social media is used passively, much like traditional media, we observe more positive public health outcomes. By contrast, active participation is associated with worse health outcomes. Thus, social media could be used as a medium for health promotion. However, public health campaigns must be aware of this reality when engaging with social media users. Future studies should further investigate social media interventions to make social media users more active in posting health-related information on social media. Moreover, they can analyze the tendency from social media consumption that could have an impact on social media users’ anxiety and fear.

## Appendix

Multimedia Appendix 1 Questionnaire.

## Abbreviations

AIC: Akaike information criterion
AVE: average variance extracted

## Data Availability

The data sets during generated or analyzed during this study are not publicly available due to the lack of authority to share data.
