# Supplementary material for: Impact of Social Media Usage on Users’ COVID-19 Protective Behavior: Survey Study in Indonesia
Source: JMIR Form Res. 2023 Apr 13;7:e46661. doi: 10.2196/46661 (PMC10141255; doi:10.2196/46661)
Supplement: Multimedia Appendix 1 [file formative_v7i1e46661_app1.docx]

# Appendix

## Survey instrument

This section presents the questions from the survey instrument used to inform the constructs used in this study. Each construct below lists the questions whose responses were used for constructing them. The questions are presented in a different order from that in which they were presented to the respondent. The reader also notices that some items initially meant to inform passive participation were excluded from analysis after data collection. Upon further inspection, we concluded that there was too substantial an overlap between the excluded items and those belonging to the active participation construct.

Trust

1. I believe that the social media platforms that I use have mechanisms in place to guarantee the trustworthiness of the information posted.
2. I trust the social media accounts that I follow.
3. I trust the information that I read on the social media accounts that I follow.
4. Overall, the social media accounts that I follow are trustworthy.

Active participation

1. I post COVID-19 content on social media.
2. I usually spend time posting COVID-19 content on social media.
3. I actively post COVID-19 content.
4. I engage in discussions on COVID-19 content.

Passive participation

1. I receive messages about COVID-19 on my social media.
2. I read messages about COVID-19 on my social media.
3. I “like” messages about COVID-19 on my social media.
4. (Excluded) I forward messages about COVID-19 on my social media.
5. (Excluded) I comment or reply to messages about COVID-19 on my social media.
6. (Excluded) I send messages about COVID-19 on my social media.

Anxiety

1. When I read the COVID-19 articles on social media, I feel nervous, anxious or on edge.
2. When I read the COVID-19 articles on social media, I cannot stop or control worrying.
3. When I read the COVID-19 articles on social media, I worry too much about different things.
4. When I read the COVID-19 articles on social media, I am trouble relaxing.
5. When I read the COVID-19 articles on social media, I am so restless that it is hard to sit still.
6. When I read the COVID-19 articles on social media, I become easily annoyed or irritable.
7. When I read the COVID-19 articles on social media, I am afraid that something awful might happen.

Self-efficacy

1. COVID-19 articles posted on social media are valuable for protection.
2. COVID-19 articles posted on social media account work for protection.
3. COVID-19 articles posted on social media are effective for protection.
4. My chance of catching COVID-19 is reduced by reading COVID-19 articles posted on social media.

Protective behavior

1. I am vaccinated against COVID-19.
2. I keep a physical distance of at least 1 meter from others.
3. I wear a properly fitted mask when physical distancing is impossible and in poorly ventilated settings.
4. I clean my hands frequently with alcohol-based hand rub or soap and water.
5. I cover my mouth and nose with a bent elbow or tissue when I cough or sneeze.
6. If I develop symptoms or test positive for COVID-19, I self-isolate until I recover.
